# Fractal nature of galaxy clustering in the updated CfA redshift catalog

**DOI:** 10.1038/s41598-026-36013-3

**Published:** 2026-01-25

**Authors:** Wiesław M. Macek, Dariusz Wójcik

**Affiliations:** 1 Laboratory for Solar System Physics and Astrophysics, Space Research Centre, Polish Academy of Sciences, Bartycka 18A, Warsaw, 00-716 Poland; 2 Institute of Physical Sciences, Cardinal Stefan Wyszyński University, Wóycickiego 1/3, Warsaw, 01-938 Poland

**Keywords:** Scaling, Multifractals, Universe, Galaxies, Clustering, Mass distribution, Astronomy and planetary science, Physics

## Abstract

We have recently argued that the expansion of the Universe is compatible not only with standard homogeneity, but also with fractal homogeneity in a hierarchical fractal cosmology. In this work we further test this paradigm using the galactic distances obtained from the Updated CfA Redshift Catalogs. We confirm that the observed multifractal spectrum is consistent with the weighted Cantor set models characteristic of turbulence in space magnetized plasmas such as the solar wind in heliosphere, the very local interstellar medium and even in laboratory experiments. The degree of multifractality is smaller than that found inside the heliosphere and shows some variations between nearby and more distant galaxies, which may be related to the presence of voids in the large-scale matter distribution. A possible asymmetry in the spectrum may be attributed to some deviations from the Hubble’s law for an ideal uniform expansion. Overall, the deviations from homogeneity revealed by multifractal analysis should be broadly consistent with $\Lambda$CDM large-scale structure formation.

## Introduction

This study examines whether the fractal scaling laws discovered through multifractal analysis offer a plausible explanation for the distribution of galaxies in the visible Universe. We demonstrate that the observed multifractal spectrum is mostly in line with the weighted Cantor model that is characteristic of laboratory and space turbulence. The universal multifractal function for galaxies resembles that observed by NASA’s Voyager missions in the outer heliosphere and even at the heliopause, the outermost heliospheric boundary.

In the eighteenth century Immanuel Kant suggested that some nebulae might be distant systems of stars, but the first galaxy beyond the Milky Way was discovered only in 1924. In fact, by the early twentieth century, based on observations using 2.5-m and 5-m telescopes on Mount Wilson and Palomar Mountain, respectively, Edwin Hubble established the view of the expanding Universe with galaxies receding from the Solar System, with velocities roughly proportional to their celestial distances. At present, after the past one hundred years, one can estimate that even a trillion galaxies, $(0.2 - 2) \times 10^{12}$, may exist in the entire Universe. Some fractions of them are now classified and well catalogued. Nevertheless, this allows us to study in more detail the large-scale structure of the distribution of galaxies in the Universe.

Incidentally, if the infinite Euclidean three-dimensional space ($D = 3$) had been filled with uniformly distributed celestial bodies and a constant density of mass distribution, this would have led to the sky always being lit near uniformly; this “Blazing Sky” effect is often called Olbers’s paradox. Alongside this the Newtonian gravitational force exerted on an object (immersed in an infinite gravitational potential) would also have been infinite^1^, p. 92. Admittedly, this paradox can be eliminated by relativistic theory and the expanding Universe.

Therefore, despite the discovery of large, massive, inhomogeneous structures with vast spatial voids – common features in astrophysical observations – the standard cosmological model, based on the theory of general relativity, still employs a similar approximation, asserting that the Universe is homogeneous, at least on sufficiently large scales, e.g.,^2^. In particular, Yadav et al. (2005) tested the assumption of cosmic homogeneity by analyzing the galaxy distribution within the Sloan Digital Sky Survey (SDSS) Data Release One (DR1)^3^, and Scrimgeour et al. (2012) investigated the transition to large-scale cosmic homogeneity using the WiggleZ Dark Energy Survey in agreement with $\Lambda$CDM N-body simulations^4^. Recently, West et al. (2025) investigated the evolution of galaxy cluster alignments, finding that their orientations are correlated over large scales (up to 200–300 comoving Mpc) and persist at high redshifts (z $\backsimeq$ 1). This suggests coherent structures in the universe’s cosmic web are larger than previously thought, and these findings are consistent with predictions from the standard $\Lambda$CDM cosmological model^5^.

Therefore, since the galaxies are actually clustered, in patches, as communicated, e.g., in Ref. ^6^, the expansion of the Universe is basically compatible not only with standard homogeneity but also with fractal features on small scales in a hierarchical fractal cosmology, as postulated by Mandelbrot^1^, ch. 32, and later proposed for inhomogeneities in the distribution of large scale structures in the Universe by various authors, e.g.^7,8^. Further, the available data satisfy power law distributions of mass with various exponents that are substantially lower than three, ranging from a value greater than 1 to about 2, see part III of his seminal book^1^. This would correspond to specific values of various fractal dimensions, $D < 3$, see the monograph^9^, ch. 3.3 and Ref.^10^, ch. 4. Naturally, this fractal approach would allow for a dark night sky for any scenario of the evolution of the Universe. Therefore, in this paper we intend to investigate whether the fractal scaling laws identified through multifractal analysis provide a reasonable explanation of the galaxy distribution in the visible Universe.

Furthermore, we have recently argued that a simple nonlinear law could possibly be important for the origin of the Universe resulting in fractal or multifractal features^9^, ch. 3.4,^10^, ch. 4. According to the standard model of the evolution of the Universe, the first stars and galaxies appeared 200–400 millions years after the Big Bang, i.e., much later than the microwave background light was emitted (400,000 years), as illustrated in Fig. 2.3 of Ref. ^9^. Apparently, the conditions of these earlier times are imprinted on this light and could possibly form a backlight for later development of the Universe. But to find a direct connection between background fluctuations and the currently observed fractal scaling laws is still far beyond the scope of the current study. Nevertheless, the fractal view of galaxy clusters is supported by luminous radiation data and is consistent with a flat Universe in thermodynamic equilibrium; in addition, this certainly satisfies the Copernican principle.

Some simple monofractal methodology for distributions of galaxies as fractal systems have recently been discussed in the astrophysical literature by Teles et al. (2021, 2022)^11,12^ and references therein, including a correlation dimension calculated to probe homogeneity in the local Universe^13^. However, it seems that the clustering structures with number *N*(*l*) at distance *l* are better explained by the multifractal spectrum of dimensions $f(\alpha )$ with $N(l) \propto l^{-f(\alpha )}$, especially for nonlinear systems in which different parts of the available phase space are visited with varying probabilities [e.g.^14,15^]. The richness of various fractal scaling behaviors has been exploited in Ref. ^16^. Traditional methods to study fractal properties of the Universe were discussed in Chapter 4 of the book by Vicent Martínez and Enn Saar “Statistics of the Galaxy Distribution” (2002)^17^. In this paper we apply our novel methods to study the fractal character of the distribution of galaxies, developed and successfully used in the study of the magnetospheres and of the Sun’s heliosphere. After early testing of fractal features of the solar wind plasma^18^, this method has been successfully verified experimentally in a plethora of space missions near the Sun^19–22^ (as more recently analyzed even on very small kinetic scales in Solar System’s plasmas, e.g.^23–27^).

Interestingly, the universal multifractal function for galaxies is similar to that identified by NASA’s Voyager missions in the outer heliosphere [see^20,21,28^] and even at the heliospheric boundaries by Macek et al. (2014) [see^22^]. Since the multifractal spectrum is expected to exhibit some universal properties [e.g.,^29^], we therefore apply similar fractal numerical methods here for the direct determination of the multifractal spectrum of the distribution of galaxies on cosmological scales, using the best currently available catalog [see, e.g.,^30^]. We show that the observed multifractal spectrum is basically consistent with the weighted Cantor model characteristic of turbulence in space and laboratory experiments^22,31,32^.

In Section Galactic data, a consistent description of the best currently available Updated Redshift Catalog (*UZCAT*) of the observed galaxies is provided, while Section Fractal analysis outlines modern tools of multifractal analysis (with the multifractal model in Subsec. Multifractal model). The vital results of our analysis are presented in Section Results, which demonstrates that the solutions of the weighted Cantor models are in good agreement with the observed multifractal spectrum of the galaxy distribution. Finally, Section Conclusions emphasizes the significance of the identified fractal scaling laws, which could be an important contribution toward the ultimate explanation of the distribution of matter in the visible Universe.

## Galactic data

We have used in our analysis the redshift data obtained from the Smithsonian Astronomical Observatory Telescope Data Center, available from http://tdc-www.harvard.edu/zcat/velocity.dat. Instead of the older *CfA* catalog with only 359 objects and the apparent magnitudes $m \le 14.5$, as analyzed in Ref. ^33^, we have now examined the Updated (June 2008) CfA Redshift (*Z*) CATalog (*UZCAT*) compilation up to about one million (from a total of a trillion) various observed galaxies, see http://tdc-www.harvard.edu/zcat/zcom.htm. This catalog originally consisted of various sets of galaxies (e.g., NZ40, SDSS, 2dF, 6dF, and ZCAT), and later other published observations of some galaxies were added by the catalogue authors, e.g.,^34–36^, including *ZBIG* responsible for higher relativistic velocities $>100,000$ km $\hbox {s}^{-1}$, cf.^37^. However, we have not used velocities with negative source designations (19,517 observations), which are in the private domain (and hence cannot be used without the owner’s consent).

After all, the data assembled by various authors for studying the large-scale structure of the Universe are basically complete in terms of redshift information, but not necessarily for some other properties such as diameter, magnitude, and references. As is known in statistics, data completeness is a measure of how much essential information is included in a dataset or a model, and describes whether there are any gaps, missing values, or biases introduced impacting the results. This property is obviously important, as analysis based on incomplete data is not meaningful, and the results may be questionable. It can be tested in various ways, for instance by calculating the percentage of completeness for individual subsets and the entire dataset, or by visualizing the distribution and structure of missing data and testing / comparing distributions. In our case, as discussed in Appendix the merged UZCAT sample is sufficiently complete for our study. However, for individual smaller sets the percentage of completeness is around 85-95%, which is certainly acceptable, with the lowest completeness in the CfA survey at only $\sim$80%. For the whole set, which is arguably large, we have systematically used a random data sampling method to estimate completeness, and the results were statistically robust against subsampling.

Hence, the velocities based on the redshift data are the best available with respect to the reported measurement errors and source reliability, the primary, purpose of this catalog is to be a complete list of galaxies with radial velocities for mapping and statistical studies. Incidentally, following the recommendation that users should remove objects of type $> 20$, which were misclassified as galaxies, before using this galaxy catalog, 14,177 observations of $V_H$ have been omitted. The most frequent type was 25 – a plate flaw, stars, and other misclassifications.

We have used here only the radial velocities $V_H(r) < c$, with the speed of light *c* = 299 792 458 m $\hbox {s}^{-1}$, for a relativistic redshift $z = \sqrt{\frac{1 + V_H/c}{1 - V_H/c}} - 1$, see e.g.^38^, which in the nonrelativistic limit of $V_H \ll c$ reduces to $z \approx V_H / c$. The velocities can be corrected for the motion of the Sun, with an apex velocity of $\sim$230 km $\hbox {s}^{-1}$, right ascension (RA) 18 h 28 m and declination (Dec.) $+30 \deg$ (North in galactic coordinates). We have, cf.^37^

1 $$\begin{aligned} V_H = \left\{ \begin{array}{ll} c\,z & \text {for }V_H \ll c, \\ c \, \frac{(1 + z)^2 - 1}{(1 + z)^2 + 1} & \text {otherwise.} \end{array} \right. \end{aligned}$$

for the assumed standard casting cosmology. Therefore, the heliocentric distance to a galaxy under study is given by

2 $$\begin{aligned} L_H := \left\{ \begin{array}{ll} \frac{c\,z}{H_0} & \text {for }z \ll 1, \\ \frac{c}{H_0} \ln (1+z) = \frac{c}{2 H_0} \ln (\frac{1+ V_H/c}{1- V_H/c}) & \text {otherwise,} \end{array} \right. \end{aligned}$$

with a Hubble parameter (present epoch) $H_{0}$ = 70 km $\hbox {s}^{-1}$ Mpc $^{-1}$.

Strictly speaking, we have eliminated negative (blueshifted) redshifts *z*, eliminated data gaps ($\sim 50,000$ blank velocities), and removed outliers using the IQR method, which is particularly useful for skewed data (in contrast to usual Z-score method), i.e., $\text {IQR} = Q_3 - Q_1$, where $Q_{1,3}$ are the first and third quartiles respectively, and then the outliers are defined as observations below $Q_1 - 1.5\,\text {IQR}$, or above $Q_1 + 1.5\,\text {IQR}$. Thus, we have analyzed the sample of 759, 285 observations down to magnitude $m \lesssim 29.5$ (as limited by the Hubble Space Telescope) and moderate relativistic velocities up to $V_H/c \approx 1/2$, corresponding to $z \approx 0.73$). After all, one can confirm that for the currently estimated diameter of the Universe of about $2 R_\textrm{max} \approx 28.5$ Gpc, the maximum receding velocity in most remote galaxies in the last category denoted by violet should be $V_\textrm{max} = c \tanh ({2 R_\textrm{max} H_0}/{c}) = 293,018$ km $\hbox {s}^{-1}$ (with $V_H/c = 0.98$ and a very large redshift $z_\textrm{max} = 8.35$).

On the other hand, for ultra-relativistic velocities Eq. (1) should be corrected accordingly. We are also aware that using only the radial distance limits our ability to explain the three-dimensional structure of galaxy distribution. However, we believe that the identification of fractal scaling in galaxy distribution is an important step toward resolving a fundamental issue in cosmology: whether the Universe is homogeneous on large scales or exhibits fractal properties. Admittedly, more recent datasets such as SDSS DR19, DESI, and Euclid forecasts might provide more comprehensive and uniform coverage^39^, see https://www.sdss.org/dr19/bhm/programs/.

**Fig. 1:**
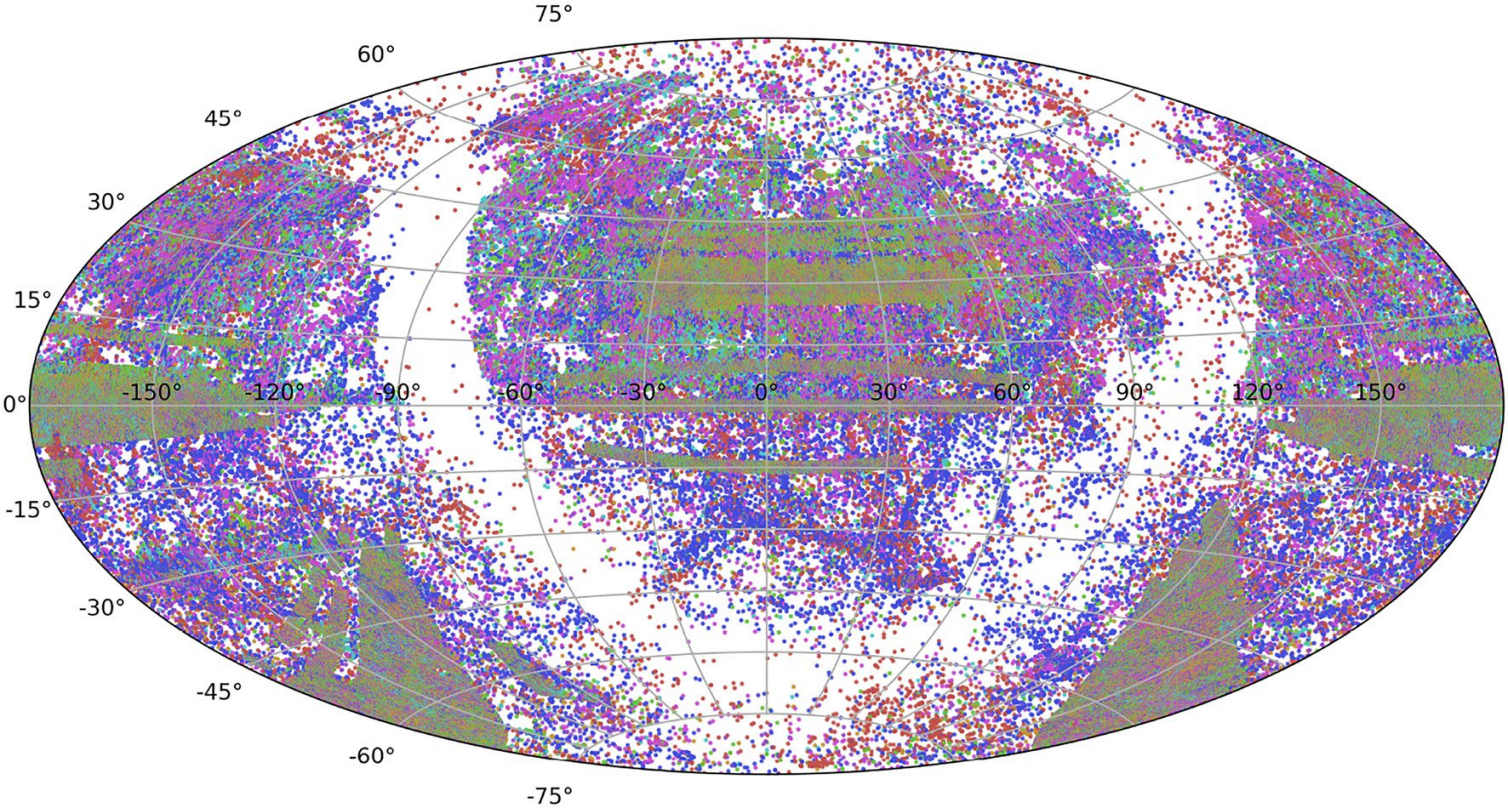
Sky map showing the distribution in different categories of galaxies: red, blue, magenta, cyan, green, orange, and violet, according to their recession velocity, based on the UZCAT updated (2008) catalog, with populations counts provided in Table 1.

The plot of the distribution on the sky of the selected galaxies from *UZCAT* (Aitoff projection) is shown in Fig. 1, for the following various categories of nearby increasingly distant galaxies: red, blue, magenta, cyan, green, orange, and violet. We have used here right ascension and declination in the Galactic (J2000) coordinate system (centred at $0^{\circ }$ increasing to the left). In particular, the green and orange groups represent the well-studied regions of the 2dF GRS (initially  100,000, increasing to  380,000 points) http://www.2dfgrs.net. The SDSS DR3 Survey https://classic.sdss.org/dr3/^40^ consists of $\sim 350,000$ galaxies. We include the LCRS and the Century surveys, extensively studied by John Huchra and Zwicky. The clusters are based on published finding charts and these clusters are standardized by ID’s using Dressler’s numbers^41^.

Apparently, the observable Universe, with possible hundreds of billions of large galaxies, is not a chaotic scatter. The galaxies form intricate filaments and other large structures, shaping a web-like pattern that defines the large-scale structure of the cosmos. This pattern reflects the behaviour of dark matter and provides insights into the Universe’s overall structure and evolution. Obviously, differences in the population of each category of galaxies could result in somewhat different fractal and multifractal characteristics. The MCAR (Missing Completely at Random) test can indirectly assess completeness or the impact of missingness, and the resulting p-value of this test is $>0.05$ in all cases, so one cannot reject the null hypothesis, suggesting that data is likely missing completely at random. Therefore, using MCAR, including MAR (Missing at Random), and MNAR (Missing Not at Random) tests^42^, we have verified that the small incompleteness of the redshift data used in our analysis does not change the obtained results, as listed in Table 1, where the population of galaxies with recorded redshifts among the galaxies in the catalogue is also provided.

**Fig. 2:**
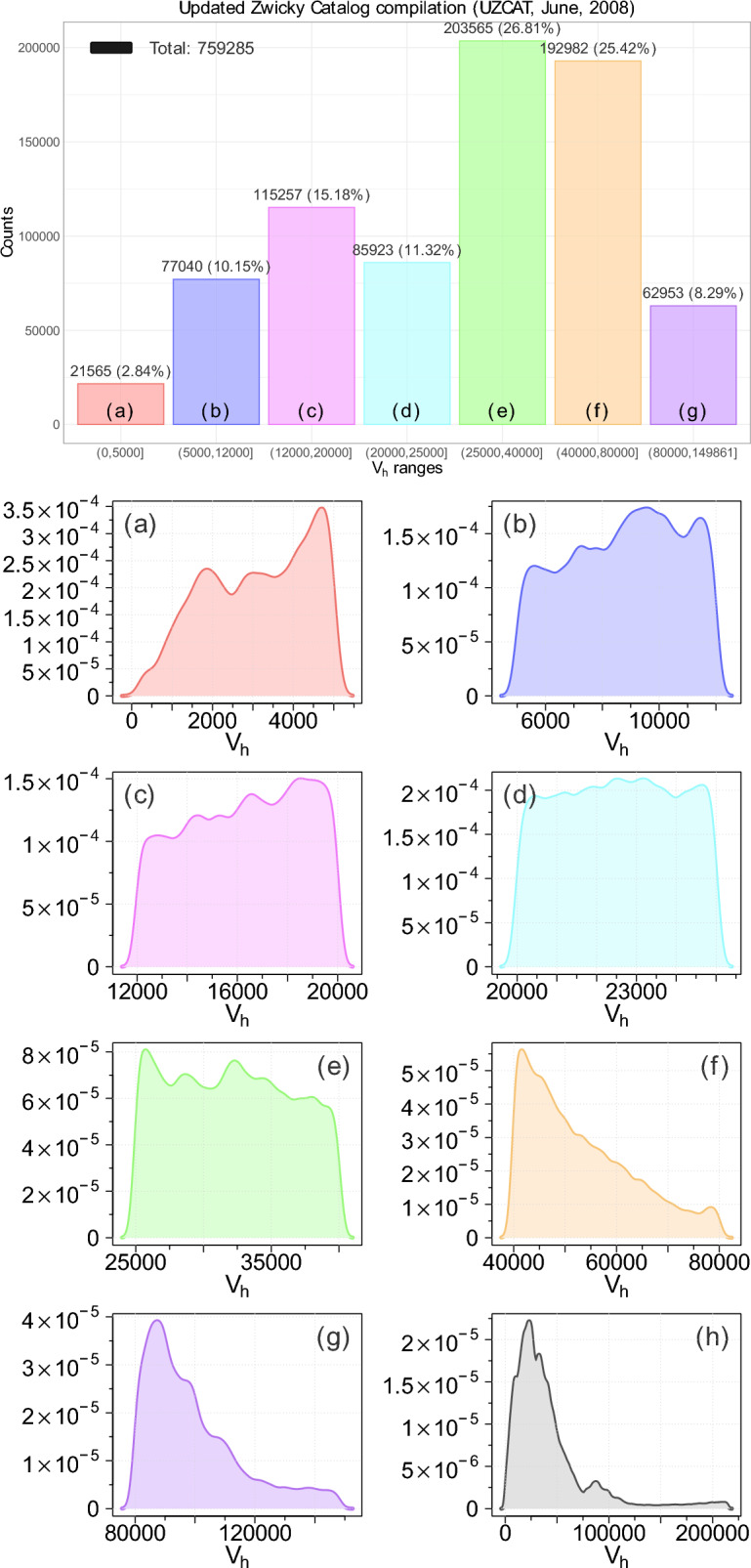
The box plots of distribution and probability density functions (PDFs) of different coloured categories of galaxies red, blue, magenta, cyan, green, orange, and violet depending of the receding speed from the UZCAT updated (2008) catalog with populations displayed in Table 1.

In Fig. 2 box plots of various populations for the following categories of the galaxies under study: red, blue, magenta, cyan, green, orange, and violet are displayed as a function of the receding speed together with the empirical probability density functions (PDFs), which have been computed using kernel density estimates (KDE). All the KDE plots generally show low densities across different ranges. They exhibit minor but no dominant peaks, indicating a multimodal distribution with several small clusters. The data points appear to be spread fairly evenly across the ranges, with no significant concentration. The skewness, however, is clearly pronounced in the contrasting cases.

## Fractal analysis

The basic concepts of fractal sets are elucidated in standard textbooks [e.g.^29,43^]. We note only that fractals are characterized by *self-similarity*, which is described by a single fractal dimension (independent of the scale *l*). On the other hand, a multifractal is a more complex object that can exhibit different self-similarities (dependent on the scale *l*), and is described by the spectrum of dimensions, or a multifractal singularity spectrum^29^, ch. 10.

### Fractal characteristics

A comparison of the main characteristics of fractals (using the usual measure of the volume of a set) and multifractals (with a probability measure describing the likelihood of visiting a fraction of the set) has been thoroughly discussed in Sec. 1 of Ref. ^22^.

As is well known, contrary to the usual monofractal scaling, basically two universal functions are characteristic for multifractals. Namely, for a set consisting of *N* elements with probability measures $p_i(l)$ associated with a given scale *l*, the generalised dimension is defined as

3 $$\begin{aligned} D_{q}=\frac{1}{q-1}\underset{l\rightarrow 0}{\lim }\frac{\log \sum _{i=1}^N (p_i)^q}{\log l}, \end{aligned}$$

while the multifractal singularity spectrum $f(\alpha )$ as a function of the singularity strength $\alpha$ ($p_{i}(l)\propto l^{\alpha _{i}}$) is defined by

4 $$\begin{aligned} f({\alpha }) = \underset{\varepsilon \rightarrow 0}{\lim }~~\underset{l\rightarrow 0}{\lim }\frac{\log [N_l(\alpha + \varepsilon ) - N_l(\alpha - \varepsilon )]}{\log 1/l}. \end{aligned}$$

In particular, for $q = 0$ one recovers a simple capacity (box-counting) dimension, $D_0 = \lim _{l \rightarrow 0} \log N / \log l$, which represents the scaling of how measures are distributed in the support of the set. Next, for $q=1$ the information dimension, $D_1~=~\lim _{l \rightarrow 0} {\sum _{i=1}^{N} [p_i(l) \log (p_i(l)]}/{\log (l)},$ with a geometrical average of $D_1~\approx ~<\log p>_\textrm{av} / \log l$ is obtained (using de l’Hôpital’s rule), while for $q=2$, the $D_2$ corresponds to the well-known standard correlation dimension $D_2 = \lim _{l \rightarrow 0} {\sum _{i=1}^{N} \log p^2_i(l)}/{\log (l)}$ with the ordinary arithmetic average $D_2 \approx \log <p>_\textrm{av}/\log ~l$, see Ref. ^44^. In general, the generalised dimensions $D_q$ are nonlinear functions of any given real index *q* and provide important information about multifractality of the system^29^. Equivalently, the universal singularity spectrum $f(\alpha )$, with the maximum value $f(\alpha _0) = D_0$, characterize multifractality of the system under study^43^. The line joining the origin to the point at $\alpha = D_ 1$, the information dimension, is tangent to the shape of the spectrum. These functions, illustrated in Figure 3.7 of Ref. ^9^, and thoroughly discussed in Refs. ^20,21^, and^44^, allow a comparison of the experimental results with the phenomenological models of turbulence^45,46^.

In addition to the usual probability measure $p_i(l)$, we can also define the following higher-order pseudoprobability measures associated with each scale *l*:

5 $$\begin{aligned} \mu _{i}(q, l) \equiv \frac{p^{q}_{i}(l)}{\sum _{i=1}^{N} p^{q}_{i}(l)}. \end{aligned}$$

Using a fractal dimension index $f_i (q, l) \equiv \log \mu _i (q, l) / \log l$), one can directly calculate the multifractal spectrum as the average of the pseudoprobability measure $\mu _i(q, l)$ according to Eq. (5) denoted here by simple brackets $\langle \cdots \rangle$^47^

6 $$\begin{aligned} f(q)~\equiv ~\lim _{l\rightarrow {0}}{\sum _{i=1}^{N} \mu _{i}(q, l)~f_i(q, l)} = \lim _{l\rightarrow {0}}~\frac{\langle \log \mu _i(q, l) \rangle }{\log (l)}. \end{aligned}$$

The average value of the singularity strength is given by^48^

7 $$\begin{aligned} \alpha (q)~\equiv ~\lim _{l\rightarrow {0}}{\sum _{i=1}^{N} \mu _{i}(q, l)~\alpha _i(l)} = \lim _{l\rightarrow {0}}~\frac{\langle \log p_i(l) \rangle }{\log (l)}. \end{aligned}$$

### Multifractal model

We have already argued that simple nonlinear or fractal models provide a useful tool for phenomenological analysis of complex turbulent media^10,49^. For example, the generalised weighted Cantor set is a simple example of multifractals, as explained e.g. in the textbook^43^. This model is illustrated in Fig. 2 of Ref. ^44^. When constructing this model with scale parameter $l_1 = l_2:= \lambda \le 1/2$ we have the analytical expressions for $D_q$ and $f(\alpha )$ [e.g.^19^]. Namely, if measures *p* and $1-p$ are applied to the left and right remaining parts of a unit interval the function $\tau (q) \equiv (q-1) D_q$ is given by Equation (11) in Ref.^21^

8 $$\begin{aligned} \tau (q) = \frac{\log [p^q + (1-p)^q]}{\log \lambda } \end{aligned}$$

and for $\alpha (q) = \tau ' (q)$ we have the following formula:

9 $$\begin{aligned} \alpha (q) = \frac{1}{\log \lambda }~\frac{p^q \log p + (1-p)^q \log (1-p)}{p^q + (1-p)^q}. \end{aligned}$$

Then, using the Legendre transformation, we obtain the explicit formula for the multifractal spectrum $f({\alpha (q)}) = q \alpha (q) - \tau (q)$:

10 $$\begin{aligned} f(\alpha ) = \frac{q \left[ (1 - p)^q \log (1 - p) + p^q \log p \right] - \left[ (1 - p)^q + p^q \right] \log \left[ (1 - p)^q + p^q \right] }{\left[ (1 - p)^q + p^q \right] \log \lambda }. \end{aligned}$$

However, for a more developed generalised two-scale weighted Cantor set we must specify two scales $l_1$ and $l_2$ ($l_1 \ne l_2$), satisfying $l_{1} +l_{2} \le 1$. In this case, one needs to solve for $\tau (q)$ the transcendental equation, e.g.,^29^,

11 $$\begin{aligned} \frac{p_1^{q}}{l_{1}^{\tau (q)}}+\frac{p_2^{q}}{l_{2}^{\tau (q)}} = 1, \end{aligned}$$

which is only slightly more general than the analytical solution given by Eq. (8). Finally, it is worth mentioning that the standard middle-thirds monofractal Cantor set model is recovered only for $\lambda = 1/3$ and $p= 1/2$, with $D_0 = \ln 2 / \ln 3$.

The difference between the calculated maximum and minimum dimensions, corresponding to the regions in the phase space with the least and most dense probability densities, has been proposed in Ref. ^44^ and^19^

12 $$\begin{aligned} \Delta \equiv \alpha _{\textrm{max}} -\alpha _{\textrm{min}} = D_{-\infty } - D_{\infty } = \left| \frac{\log (1 - p)}{\log l_2} - \frac{\log (p)}{\log l_1} \right| , \end{aligned}$$

where $\Delta$ quantifies the degree of multifractality. Naturally, this parameter $\Delta$ also reflects deviations from strict monofractal self-similarity, and it can serve as a measure of intermittency, as discussed in^45^, chapter 8. Another quantitative parameter describing the multifractal scaling is the measure of asymmetry of the spectrum defined in Ref. ^19^

13 $$\begin{aligned} A \equiv \frac{\alpha _{0} -\alpha _{\textrm{min}}}{\alpha _{\textrm{max}} - \alpha _{0}}, \end{aligned}$$

where $\alpha = \alpha _0$ is the value at which the spectrum reaches its maximum, $f(\alpha _0)=D_0$. The case when $A=1$ ($l_1 = l_2 = 1/2$) corresponds to the one-scale *p*-model [e.g.,^50^].

Now, following Ref. ^51^ the probability measures *p*(*l*) associated with a given scale $l:= L_H$, as discussed in Sect. Galactic data, can be constructed directly from the observed distribution of galaxies. Specifically, one first normalizes the series of average numbers of the observed objects $n(l_i)$ in *i*-th shell of radius $l_i$, where $i = 1, \ldots , \mathcal {N} = 2^{m}$ (e.g., taking *m* = 17). For $j = 2^{m-k}$, $k = 0, 1, \ldots , m$, one defines:

14 $$\begin{aligned} p(x_{j}, l) \equiv \frac{1}{\mathcal {N}}{\sum _{i = 1 + (j-1)\Delta l}^{j \Delta l} n(l_i)} = p_j (l), \end{aligned}$$

where the successive average values $\langle n(l_i + \Delta l) \rangle$ are taken over the intervals between $l_i$ and $l_i + \Delta l$, for each $\Delta l = 2^k$ with the total $\mathcal {N}$ number of galaxies in the system [cf.^20^].

One can show that in the inertial range of scales, the average value of the *q*-th moment of *p* at various scales *l* scale as^51^

15 $$\begin{aligned} \langle p^{q} (l) \rangle \sim l^{\gamma (q)}, \end{aligned}$$

where the exponent $\gamma$ is related to the generalised dimension via $\gamma (q) = (q-1) (D_q - 1)$. Using this method the values of $D_q$ can be determined from the slopes of $\log \langle p^q(l)\rangle$ versus $\log l$ for each real *q*, as expressed in Eq. (15). Alternatively, the multifractal function $f(\alpha )$ versus scaling index $\alpha$, which characterizes the universality of the multifractal scaling behavior, can be obtained using the Legendre transformation. It is worth noting, however, that we have obtained this multifractal universal function directly from the slopes given in Eqs. (6) and (7), using this direct method in various situations [see,^19–22^].

## Results

Admittedly, with the *CfA* limited observations, one can only determine the points near the maximum of $f(\alpha )$ [cf.^33^]. One can possibly extrapolate these points near the intercepts at the maximum, $f(\alpha _0) = D_0$. On the other hand, in our study based on a much more extensive *UZCAT* dataset of redshifted distances presented in Sect. Galactic data, Eq. (2), and using the fractal methods described in Section Fractal analysis with the multifractal model of Section Multifractal model, we are now able to obtain a more reliable multifractal spectrum of the distribution of galaxies in the Universe.

Therefore, we first consider astronomical surveys at different right ascension (RA) and declination (Dec) values, as shown in Fig. 1. However, instead of plotting observations by their exact positions on the celestial sphere (which would not be exactly insightful), we first illustrate how a given property varies as a function of RA. We use this variable as a proxy for time in a series of heliocentric velocities for individual galaxies, treating the $0-24$ h range of RA (similarly to a 24-hour time period), but now expressed in the J2000 galactic frame of reference. This plot created using the right ascension (celestial equivalent of longitude) variable is commonly used in observational astronomy when tracking the position of celestial objects over time. Obviously, this leverages the regular rotation of the Earth to map RA values to observational time, assuming that the observations are evenly distributed.

**Fig. 3:**
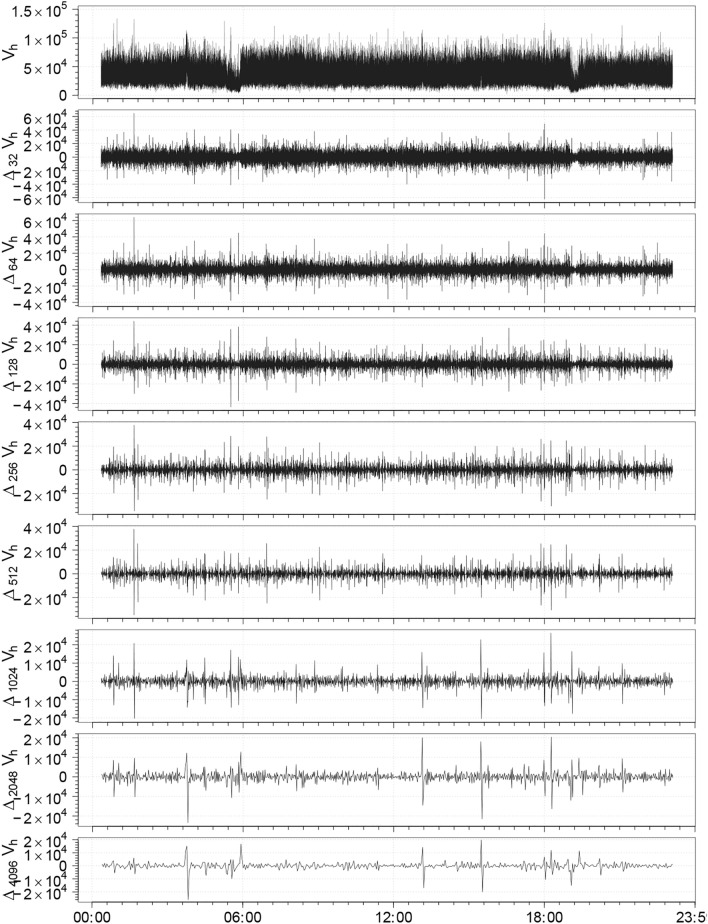
The differences of successive ${2^m}$-step averages, $\Delta _{2^m} V_{H}$ [km $\hbox {s}^{-1}$], represent large-scale speed fluctuations for $m = 5, \ldots , 12$ calculated from the observed distribution of galaxies based on the selected *UZCAT* data.

In this way, Fig. 3 displays the differences of successive $2^m$-step averages of large-scale fluctuations in the receding redshifted speeds $\Delta _{2^m} V_{H}$ (in km $\hbox {s}^{-1}$) for $m = 5, \ldots , 12$, see Sect. 9.4.2 in Ref. ^51^. One can identify patterns or trends that may correspond to certain celestial regions or astronomical phenomena. Moreover, any deviations from the ideal linear Hubble law can provide insights into large-scale structures, peculiar motions, and evolutionary effects. In particular, we observe some irregular bursty, spiky, inhomogeneous (aperiodic, and asymmetric) features of varying widths, which are characteristic for multifractal fluctuations for intermittent turbulence. In most cases, the magnitudes of positive fluctuations are somewhat greater than those for the negative fluctuations. Because time series for larger scales are magnified parts of the time series for the velocity increments for smaller scales, it seems that the cosmological fluctuations are self-affine across different scales. Hence, we can proceed with the multifractal analysis for various *q* values and scales $l:= L_H$ as defined in Section Galactic data, Eq. (2). The normalized probability measures *p*(*l*) depending on scale $l:= L_H$ are now constructed according to Eq. (14) for each category, as obtained using the *UZCAT* galaxy catalog data shown in Fig. 1.

**Fig. 4:**
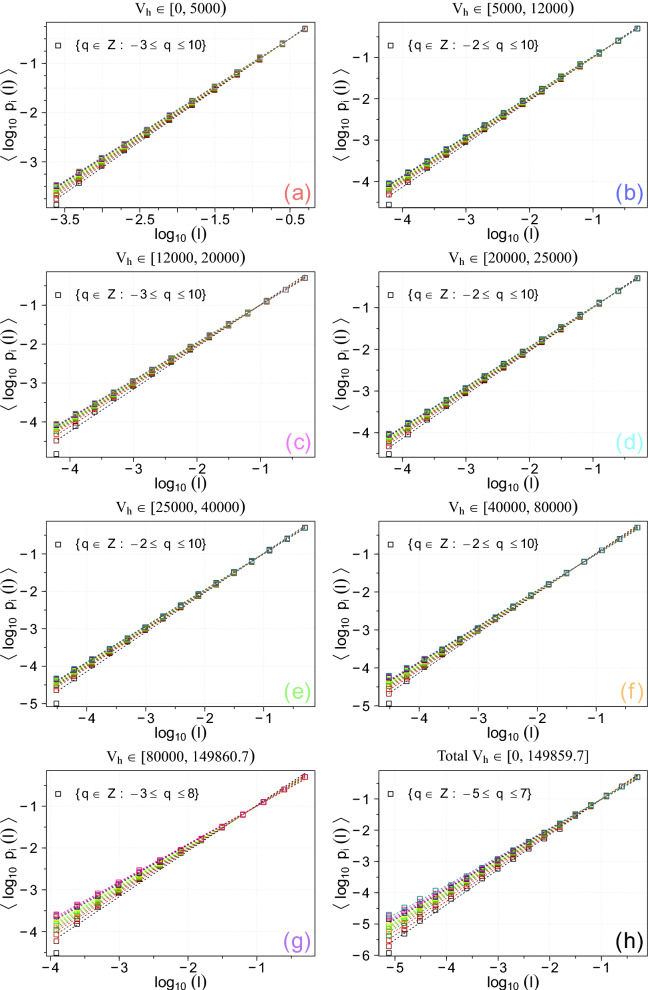
Generalised average logarithmic probability, $\langle \log _{10} p_i(l)\rangle$, (a) as a function of $\log _{10} l$ for various *q*. These results are obtained using the *UZCAT* catalog.

**Fig. 5:**
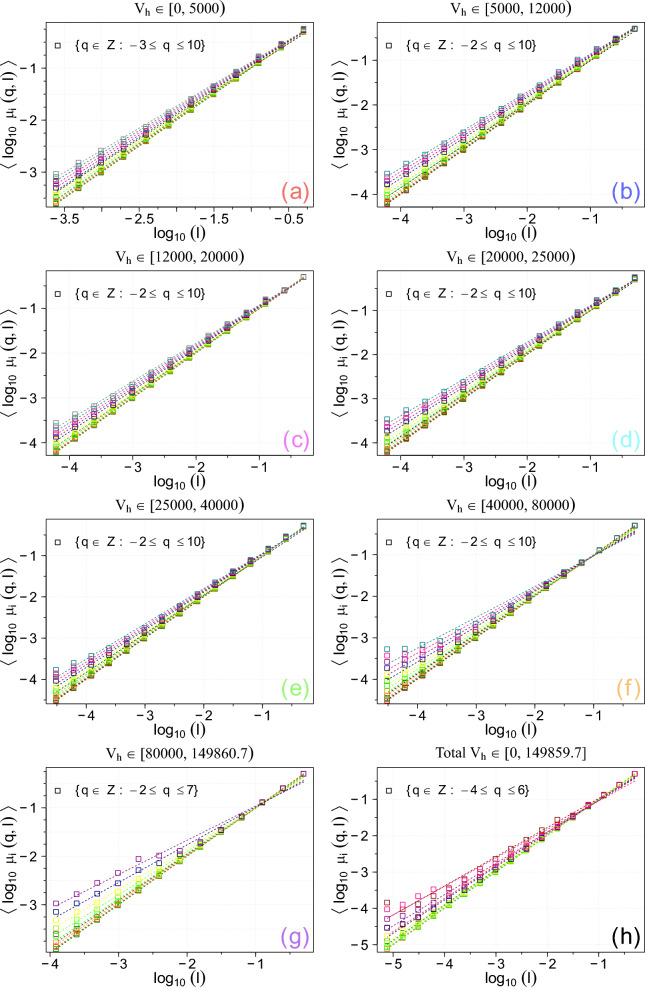
Generalised average logarithmic pseudoprobability, $\langle \log _{10} \mu _i(q, l)\rangle$, as a function of $\log _{10} l$ for various *q*. These results are obtained using the *UZCAT* catalog.

Second, in Figs. 4 and 5 both average logarithmic probability and pseudoprobability measures $\langle \log _{10} p_i(l)\rangle$ and $\langle \log _{10} \mu _i(q, l)\rangle$ versus $\log _{10} l$ for all coloured categories in the *UZCAT* catalog are now presented for the following values of $q \in [-4, 6] \cap \mathbb {Z}$ values of *q* featuring very robust fittings with $R^2 < 0.975$ and $r < 0.975$ – where *r* denotes the Pearson correlation coefficient – have been excluded. As seen, the calculated slopes can be fitted to linear straight lines over a range of scales spanning approximately 4 to 5 orders of magnitude. Hence, similarly as for the heliospheric plasma cf.^19,21,22^, we can derive the multifractal spectrum using *UZCAT* data and compared the observational points with the weighed one-scale or the two-scale Cantor set models, as discussed in Section Multifractal model.

**Fig. 6:**
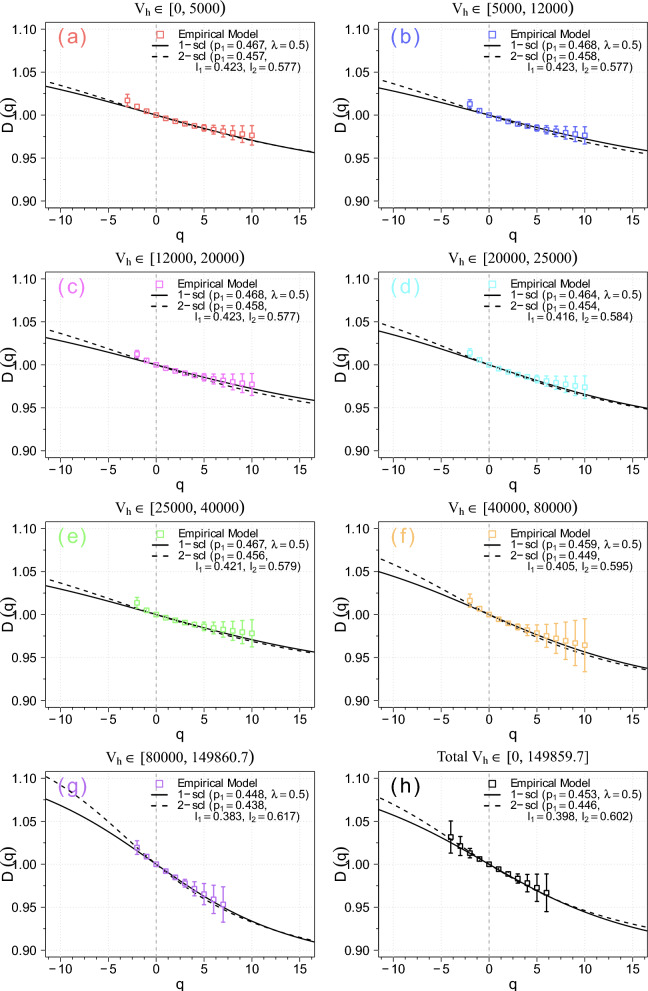
The obtained generalised dimensions $D_q$ as functions of *q* (boxes) for the observation categories (**a**-**h**) in the *UZCAT* catalog are compared with the weighted Cantor models: one-scale (continuous lines) and two-scale (dashed lines).

**Fig. 7:**
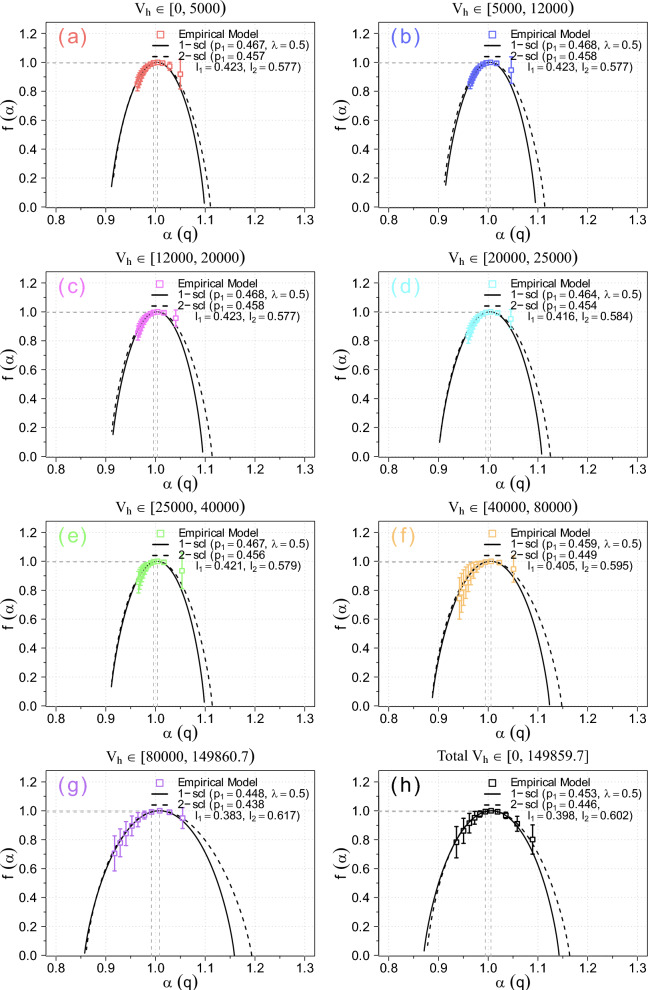
The obtained multifractal measures of the multifractal spectrum $f(\alpha )$ as function of the singularity strength $\alpha$ (boxes) for the observation categories (**a**-**h**) in the *UZCAT* catalog are compared with the weighted Cantor models: one-scale (continuous lines) and two-scale (dashed lines).

The generalised dimensions $D_q$ as a function of *q* and the universal singularity spectrum $f(\alpha )$ as functions of singularity strength $\alpha$ are displayed in Fig. 6 and 7, respectively. The values of $D_{q}$ and $f(\alpha )$, as given in Eqs. (6) and (7), are calculated using the *UZCAT* data (denoted by boxes) and compared with both Cantor set models [cf. 44, Fig. 2].

As expected, the normalized generalised dimension $D_q$ (1D proxy for normalized probability measure, quantifying multifractality) is a decreasing function of *q* and the multifractal spectrum $f(\alpha )$ is a universal concave down function of singular index $\alpha$^29^, Fig. 9.1. In particular, we have $f(\alpha _0):= D_0 = 1.0$ and $D_1 = 0.994 \pm 0.007$ and as well as $D_2 = 0.983 \pm 0.013$. It is worth noting that, after removing the normalization, the entire spectrum of $D_q$ for any real *q*, as presented in Fig. 6, is consistent with a robust estimate of 3D proxy $D_2 (r) + 2$, which reaches a value of 2.97 (1% from homogeneity) in the Local Universe ($z < 0.2$) obtained from the *SDSS* catalog, as the scale *r* increases when the transition to homogeneity scales occurs (see Fig. 5 of ref.^13^). This should, on the other hand, be compared with the values obtained for specific single fractal dimensions *D* obtained by Teles et al. (2022), who tried to challenge he standard model using different galaxy samples and somewhat higher redshifts ($z < 1$)^12^.

Here, however, we use the *UZCAT* catalogued observations, which are reasonably well consistent with the *p*-model, or one-scale Cantor set symmetric spectrum (continuous lines), fitted to the theoretical solutions of Eq. (8), and given in Eqs. (9) and (10), especially for $q > 0$ (left part of the spectrum) while for $q<0$ (right part) the agreement is somewhat less clear. By using surrogate data tests, it has already been verified that the most popular correlation dimension for the solar wind is not merely an artifact of data selection^18^, Fig. 8. A similar test for the plethora of galaxy catalogs is deferred to future detailed studies.

Naturally, an even better agreement is observed with the asymmetric two-scale (dashed lines) Cantor set model, with the corresponding parameter *p* (or $p_1 = p$, and $p_2 = 1 - p$) and lengths $l_1$ and $l_2$ given by the theoretical model in Eq. (11). Hence, the empirical values are in a good agreement with the theoretical model^9^. To correctly select all these model parameters ($p_1$, $p_2$, $l_1$, $l_2$), we have used the loss metric to find the best possible fits^52^. The method combines the MSE and MAE metrics, giving a better loss function that is less sensitive to outliers, e.g., due to irregular intervals in the time series. Furthermore, for the two-scale Cantor model (as well as for the one-scale model), we have $p_1 + p_2 = 1$ (see also Ref. ^9^), meaning that the fragmentation with probability $p_1$ for a fragment of length $l_1$ is virtually equivalent to fragmentation with probability $p_2$ for a fragment of length $l_2$. To accelerate computations, parallel processing was employed, utilizing multiple processor cores simultaneously.

However, the total degree of multifractality $\Delta \approx 0.15$ is substantially smaller than that inside the heliosphere $\Delta = 0.3-0.7$, but larger than that in non multifractal ($\Delta \approx 0$) case of the very local interstellar medium (VLISM) after the crossing of the heliopause (at $\sim 122$ AU) by Voyager 1 in 2012^22^. This suggests a predominantly simple linear fractal scaling of galaxy distribution. Admittedly, we are still able to examine only a small fraction of all the galaxies existing in the Universe. Therefore, we cannot definitely determine whether the actual distribution is close to a true fractal. Nonetheless, since the calculated correlation dimension $D_2$ is consistent with the value in the Local Universe using the *SDSS* catalog, when the transition to homogeneity scales occurs^13^, it seems that the deviations from homogeneity revealed by the multifractal analysis should be roughly consistent with $\Lambda$CDM large-scale structure formation. The parameters $p \approx 0.45$ and $\lambda = \frac{1}{2}$ for the one-scale model likely reflect the presence of voids in the large-scale matter distribution. In particular, the slightly asymmetric spectra with $A = 0.5 - 2.0$ in the two-scale weighted Cantor set model ($A \ne 1$) may be related to the deviation from the Hubble’s law for in an otherwise uniformly expanding Universe.

**Table 1:** Values of Parameters Describing Multifractality $\Delta$ and Asymmetry *A* of the Spectra for the Redshifts from the *UZCAT* Catalog for Variously Populated Categories of Distances to Remote Galaxies (in $10^3$ km $s^{-1}$).

Galactic category	Velocity max	Redshift max	Population	Multifractality	Asymmetry A
				$$\Delta$$	
Red	5	0.0168	21,556	0.0862	0.8817
Blue	12	0.0409	77,026	0.0822	0.9677
Magenta	20	0.0667	115,233	0.1225	0.4774
Cyan	25	0.0871	85,905	0.0855	1.1093
Green	40	0.1434	203,561	0.0873	0.7793
Orange	80	0.3214	192,982	0.1087	1.4238
Violet	<150	0.7321	62,562	0.1367	1.9697
Total			759,285	0.1532	0.8349

We have also calculated the multifractal parameter $\Delta$ and asymmetry *A* from Eqs. (12) and (13) for the observed Universe, as a function of distances for all categories: red, blue, magenta, cyan, green, orange, and violet. The results are presented in Table 1. The differences listed in Table  1 vary slightly, from 0.09 for nearby galaxies ($\Delta \simeq 0.1$) to $\Delta \simeq 0.14$) for the most remote galaxies receding from our Solar System. This variation likely reflects differences in the populations of receding galaxies across categories and distances. The parameters $p \approx 0.45$ and $\lambda = \frac{1}{2}$ for the one-scale model are apparently related to some voids in the large-scale matter distribution. Moreover, a possible asymmetry ($A = 0.8$) of the total spectrum for the two-scale weighted Cantor set ($A \ne 1$) could be attributed to some deviations from the Hubble’s law in an ideally uniform expanding Universe.

### Presentation

Submitted to Galaxies collection of Scientific Reports, July 2025.

## Conclusions

Based on a sample consisting of various categories of about 750, 000 galaxies taken from the *UZCAT* catalog, as highlighted by colours in Fig. 1, we have studied the large-scale distribution of galaxies in the Universe by analysing intermittent self-affine multifractal fluctuations in the average heliocentric (relativistic redshifted) velocities, as presented in Fig. 3.

Basically, using the calculated slopes depicted in Figs. 4 and 5 along with the one-scale or two-scale weighted Cantor set models, we have finally obtained the generalised dimensions and the universal multifractal spectrum shown in Figs. 6 and 7. It is worth noting that the observed multifractal spectrum is simply based on direct comprehensive analysis of redshifted distances from the best currently available catalog of observed galaxies. In this way, we have provided new important supporting evidence that the large-scale galaxy distribution most probably has a multifractal structure consistent with the weighted Cantor set model.

Because of the differences in population of various classes of galaxies, the degree of multifractality $\Delta$ of the spectrum somewhat varies between 0.09 and 0.14 for increasingly remote receding distances, as listed in Table 1, However, the degree of multifractality is rather small, $\Delta \lesssim 0.15$, being obtained from admittedly a tiny fraction of all possibly existing galaxies. Hence, one is still not able to give any definitive answer whether the galaxies in the entire Universe actually exhibit multifractal or even a simple fractal distribution, as has already been suggested in Ref. ^1^. Possible deviations from the Hubble law may be reflected in an asymmetric multifractal spectrum. We also suggest a link between multifractal characteristics and voids in the large-scale structure.

Admittedly, further investigations including 3-D simulations are needed to confirm the actual distribution of galaxies. Nevertheless, on the hundredth anniversary of the discovery of the first galaxy beyond the Milky Way, we are still hoping that the identification of fractal scaling laws of galaxies could be an important contribution to ultimate explanations of the distribution of matter in the Universe.

## Supplementary Information

Supplementary Information.

## Data Availability

The data supporting the results in this article are available through the Smithsonian Astronomical Observatory Telescope Data Center available from http://tdc-www.harvard.edu/zcat/velocity.dat.
