# Author Correction: Fractal nature of galaxy clustering in the updated CfA redshift catalog

**DOI:** 10.1038/s41598-026-59578-5

**Published:** 2026-07-06

**Authors:** Wiesław M. Macek, Dariusz Wójcik

**Affiliations:** 1 Laboratory for Solar System Physics and Astrophysics, Space Research Centre, Polish Academy of Sciences, Bartycka 18A, Warsaw, 00-716 Poland; 2 Institute of Physical Sciences, Cardinal Stefan Wyszyński University, Wóycickiego 1/3, Warsaw, 01-938 Poland

Correction to: *Scientific Reports* 10.1038/s41598-026-36013-3, published online 25 January 2026

The original version of this Article contained errors in the Galactic data section.

As a result, in the Galactic data,

"Thus, we have analyzed the sample of 783, 152 observations down to magnitude $m \lesssim 29.5$ (as limited by the Hubble Space Telescope) and moderate relativistic velocities up to $V_H/c \approx 1/2$, corresponding to $z \approx 0.73$).”

now reads:

"Thus, we have analyzed the sample of 759, 285 observations down to magnitude $m \lesssim 29.5$ (as limited by the Hubble Space Telescope) and moderate relativistic velocities up to $V_H/c \approx 1/2$, corresponding to $z \approx 0.73$).”

The original Article has been corrected.

