# Supplementary material for: Fractal nature of galaxy clustering in the updated CfA redshift catalog
Source: Sci Rep. 2026 Jan 25;16:6181. doi: 10.1038/s41598-026-36013-3 (PMC12905246; doi:10.1038/s41598-026-36013-3)
Supplement: Supplementary file 1 — Supplementary Information. [file 41598_2026_36013_MOESM1_ESM.pdf]

Appendix: Limitations and completeness of the observational data

The integrated CfA redshift compilation (UZCAT) explicitly incorporates many surveys, including SDSS DR1/DR3, 2dF, 6dF, LCRS, IRAS/PSCz, other smaller surveys, including ZBIG, and even private source entries (which have been removed). The velocities in the file are stored as heliocentric $c \cdot z$, which were subsequently converted to the rest frame, as stated in Equation (1).

The UZCAT catalogue has various limitations. Firstly, the different surveys contribute very different footprints (e.g., SDSS strips/plates, 2dF NGP/SGP strips). If treated as a single uniform sample one could misinterpret survey boundaries and overlapping regions. However, the author J. Huchra has already addressed the overlapping data. Additionally, we have employed the positional cross-match with a sensible tolerance depending on the original coordinate accuracy fields. We have flagged the multiplets and split/resolved them manually when needed, also following the "Comments" column. Furthermore, some contributors targeted special classes (LRGs, quasars, IRAS objects, radio galaxies, etc.). There are non-random color/AGN/IR biases in parts of ZCAT (e.g., Véron-Cetty quasar lists included).

Thus, we have identified survey sources, types, and classes for the objects (presenting categorical variables in data) and decided whether to include or exclude specialized programs for our scientific goal. Also, the UZCAT entries pull magnitudes from many systems (Zwicky m_{Zw}, SDSS r, APM b_J, etc.), with large ($\sim$ 0.3 mag) errors. This can conservatively be approached by restricting our analysis to regions with high-quality homogeneous photometry (e.g., SDSS footprint), and building volume-limited subsamples using magnitudes with small $\sigma \sim 0.02$. Alternatively, one could apply the probabilistic weight approach, which corrects the Eddington- vs. Malmquist-like scatter at the catalog-selection level. (e.g. https://www.aanda.org/articles/aa/full_html/2015/04/aa25489-14/aa25489-14.html)

As a result, the large surveys that dominate UZCAT each achieve high completeness within their design limits (for bright magnitudes and outside the Galactic plane): 2dF has $\geq 90\%$ completeness well above its faint limit and falls to $\approx 80-85\%$ at the faint edge in some fields, 2MASS XSC meets Level-1 requirements and is empirically $> 95\%$ complete for bright galaxies away from the plane, and SDSS reaches very high spectroscopic completeness for its main sample though with fiber-collision caveats. Huchra et al. (2012) report $\approx 91\%$ sky coverage for the merged efforts. Therefore, in general, the merged UZCAT sample is sufficiently complete for many large-scale/qualitative studies.
